# Identification and Analysis of *MYB* Gene Family for Discovering Potential Regulators Responding to Abiotic Stresses in *Curcuma wenyujin*

**DOI:** 10.3389/fgene.2022.894928

**Published:** 2022-04-25

**Authors:** Qiuhui Wei, Yuyang Liu, Kaer Lan, Xin Wei, Tianyuan Hu, Rong Chen, Shujuan Zhao, Xiaopu Yin, Tian Xie

**Affiliations:** ^1^ School of Pharmacy, Hangzhou Normal University, Hangzhou, China; ^2^ Key Laboratory of Elemene Class Anti-Cancer Chinese Medicines, Engineering Laboratory of Development and Application of Traditional Chinese Medicines, Collaborative Innovation Center of Traditional Chinese Medicines of Zhejiang Province, Hangzhou Normal University, Hangzhou, China

**Keywords:** MYB family, phylogeny analysis, expression pattern, transcriptional activation, C. wenyujin

## Abstract

*MYB* superfamily is one of the most abundant families in plants, and plays critical role in plant growth, development, metabolism regulation, and stress response. *Curcuma wenyujin* is the main source plant of three traditional Chinese medicines, which are widely used in clinical treatment due to its diverse pharmacological activities. In present study, 88 *CwMYBs* were identified and analyzed in *C. wenyujin*, including 43 *MYB*-related genes, 42 *R2R3-MYB* genes, two *3R-MYB* genes, and one *4R-MYB* gene. Forty-three MYB-related proteins were classified into several types based on conserved domains and specific motifs, including CCA1-like type, R-R type, Myb-CC type, GARP-like type, and TBR-like type. The analysis of motifs in MYB DBD and no-MYB regions revealed the relevance of protein structure and function. Comparative phylogeny analysis divided 42 R2R3-MYB proteins into 19 subgroups and provided a reference for understanding the functions of some CwMYBs based on orthologs of previously characterized MYBs. Expression profile analysis of *CwMYB* genes revealed the differentially expressed genes responding to various abiotic stresses. Four candidate *MYB* genes were identified by combining the results of phylogeny analysis and expression analysis. *CwMYB10*, *CwMYB18*, *CwMYB39*, and *CwMYB41* were significantly induced by cold, NaCl, and MeJA stress treatments. *CwMYB18* and *CwMYB41* were proved as regulators with activity of transcriptional activation, whereas *CwMYB39* and *CwMYB10* were not. They may participate in the response to abiotic stresses through different mechanisms in *C. wenyujin*. This study was the first step toward understanding the *CwMYB* family and the response to abiotic stresses in *C. wenyujin*.

## Introduction

*Curcuma wenyujin* Y.H. Chen et C. Ling is a member of the *Zingiberaceae* family and mainly cultivated in Wenzhou City, Zhejiang Province, P. R. China. Because of their various pharmacological activities, the dried rhizomes and root tubers of *C. wenyujin* have been used as traditional Chinese medicine (TCM) for over 1,000 years (Zhai et al., 2019; Li et al., 2021). Drought, high salt, extreme temperatures, and other stresses all impact the yield and quality of *C. wenyujin*. The v-myb avian myeloblastosis viral oncogene homolog (MYB) superfamily is one of most significant transcription factor (TF) families in plants, which regulates plant growth, development, and a variety of physiological/biochemical processes (Cao et al., 2020; Wang et al., 2021; Xiao et al., 2021).

There is a conserved DNA binding domain (DBD) in MYB proteins, made up of 1–4 imperfect amino acid sequence repeats i.e. “R”. There are approximately 52 amino acids in each “R”, forming a very similar folding architecture with three well-defined α-helixes. The helix-turn-helix hydrophobic core was formed by the second and third helices of each “R” with three regularly spaced tryptophans (W) or other hydrophobic residues. *MYB* superfamily is divided into four subfamilies based on the number of “R” in MYB DBD: *MYB*-related subfamily gene with a single or partial “R”, *R2R3-MYB* subfamily gene with “R2” and “R3”, *3R-MYB* subfamily gene with “R1”, “R2”, and “R3”, as well as *4R-MYB* subfamily gene with four “R1/R2” (Dubos et al., 2010).

The *R2R3-MYB* subfamilies from *Arabidopsis thaliana* and *Oryza sativa*, the model plants for dicotyledons and monocotyledons respectively, have been well identified and characterized (Dubos et al., 2010; Katiyar et al., 2012). Many R2R3-MYBs were reported to regulate the primary and secondary metabolism, cell fate and identity, developmental processes, and response to biotic, and abiotic stresses (Li X. et al., 2019; Nguyen et al., 2019; Cao et al., 2020; Xiao et al., 2021). For example, Arabidopsis MYB30 acts as a critical negative regulator promoting PIF4 and PIF5 protein accumulation in the light to regulate photomorphogenic development (Yan et al., 2020). AtMYB111 plays a role as a positive regulator in salt stress response, depending on its regulation on flavonoid synthesis by activating the transcription of chalcone synthase (*CHS*), flavanone carboxylase (*F3H*), and flavonol synthase 1 (*FLS1*) (Li B. et al., 2019). In rice, MORE FLORET 1, an MYB transcription factor, interacts with TOPLESS-RELATED PROTEINs (TPRs) proteins to regulate the organ identity and spikelet determinacy by repressing the expression of downstream target genes (Ren et al., 2020). A rice R2R3-MYB (OsC1) transcriptional regulator helps to ameliorate oxidative stress in plants by increasing anthocyanin accumulation (Upadhyaya et al., 2021). Besides, more and more *R2R3-MYB* genes were identified and characterized in other plants. In *Rosa multiflora*, RmMYB108 is positively involved in response to cold, freeze, high salt, and drought stresses. *RmMYB108* overexpressed in Arabidopsis improves the cold tolerance by reducing plant damage and promoting plant growth (Dong et al., 2021). CmMYB15 regulates the biosynthesis of lignin to enhance the resistance of chrysanthemum to aphids (An et al., 2019). *PlMYB108* from *Paeonia lactiflora* overexpressed in tobacco plants increases the flavonoid accumulation, antioxidant enzyme activities, and photosynthesis to confer drought tolerance (Wu et al., 2021).

*MYB*-related subfamily can be divided into several types based on the different structure of “R” in MYB BDB: CCA1-like type, R-R type, TRF-like type, TBP-like type, I-box like type, CPC-like type, GARP-like type, and so on (Du et al., 2013). CCA1-like MYB in MYB DBD contains a conserved “R” with the motif SHAQK (y/f)F. Rice CIRCADIAN CLOCK ASSOCIATED1 (OsCCA1) positively regulates expression of *TEOSINTE BRANCHED1* (*OsTB1*), *DWARF14* (*D14*), and *IDEAL PLANT ARCHITECTURE1* (*IPA1*) to repress tiller-bud outgrowth (Wang et al., 2020). Moreover, CCA1-like MYB protein GmABAS1 enhances abscisic acid (ABA) sensitivity by acting as a transcriptional repressor of the target gene *ABI* in ABA signal pathway (Ku et al., 2020). TBP-like MYBs contain a conserved motif LKDKW(R/K) (N/T) in “R”. Most of the known TBP-like genes encode telomere-binding proteins (Du et al., 2013). The Myb-CC type MYBs have an “R” with the SHAQK (y/f)F motif in MYB BDB and the Myb_CC_LHEQLE domain (pfam14379) in no-MYB region (Gu et al., 2022). ZmMYB-CC10 enhances tolerance to drought stress by directly activating *ZmAPX4* expression, thereby reducing H_2_O_2_ content (Zhang et al., 2022). SlPHL1 is a newly identified MYB-CC TF from tomato, which participates in Pi-starvation signaling by directly upregulating the *PSI* genes to elevate root hair growth, promote APase activity, and favor Pi uptake (Zhang et al., 2021). MYB DBD of GARP-like protein contains a conserved “R” with the motif SHLQK/MxR. There are two viewpoints about GARP-like proteins: 1) those that belong to *MYB*-related subfamily due to a similar motif but have distant relationships; and 2) those that do not belong to *MYB*-related subfamily due to the only “W” residue in conserved MYB DBD (Du et al., 2013; Safi et al., 2017). Arabidopsis overexpressing *GhGLK1* (a *GARP*-like gene) showed more adaptability to drought and cold treatments with the less leaf damage and lower ion permeability (Liu et al., 2021). Interestingly, R-R type MYB-related proteins were the exception, with two far apart “R” located in the N-terminal and middle of the sequence, respectively. The second “R” in middle of the sequence contains a SHAQK (y/f)F motif similar to that in CCA1-like MYBs. In Arabidopsis, *AtDIV2*, encoding an R-R type MYB TF, plays negative role in salt stress and is required for ABA signaling (Fang et al., 2018). In addition, *3R-MYB* and *4R-MYB* subfamilies are the smaller classes that often contain a few members in most plants.

Because of the roles of *MYB* genes in plant growth, development, metabolism regulation, and stress response, it is of great significance to study *MYB* family in *C. wenyujin* and its expression patterns in different tissues and under different stresses. With transcriptome sequence data of *C. wenyujin*, *MYB* family was systematically identified and analyzed including gene classification, evolutionary relationship, conserved domain, and motif composition for the first time. The spatial and temporal expression profiles were also analyzed, as well as the differential expression profiles of *MYB* genes in response to methyl jasmonate (MeJA), cold, and salt stresses. The findings will provide a comprehensive understanding of *MYB* family, and lay the theoretical groundwork for investigating *MYBs’* potential roles in *C. wenyujin*.

## Materials and Methods

### Plant Materials and Stress Treatment

*C. wenyujin* was used as the plant material in this study. The germplasm was obtained from Wenzhou City, Zhejiang Province, P. R. China (14 m altitude, 27°47′N, and 120°37′E). The original plants and their macroscopic characteristics were authenticated by Professor Zengxi Guo, who works at the Institute of Food and Drug Control in Zhejiang, China. Seedlings were cultured in Murashige and Skoog (MS) solid medium in an incubator (12 h light/12 h dark cycle at 22°C) for 1 month to trefoil stage to perform the expression analysis of *MYB* genes with abiotic stresses. Seedlings were cultured with 200 mM NaCl solution as salt stress treatment for 24 h. For MeJA treatment, seedlings were cultured in solution containing 250 μM MeJA for 24 h. For cold stress treatment, seedlings were cultured in an incubator at 4°C for 24 h. All samples were collected at the time points (0, 1, 3, 6, 12, and 24 h), frozen in liquid nitrogen, and then stored at -80 °C for subsequent RNA extraction.

### Identification of MYBs in *C. wenyujin*

The Hidden Markov Model (HMM) profile of MYB DBD (Accession No. PF00249) was downloaded from the Pfam database (http://pfam.xfam.org) and used as a query to identify *CwMYBs* in the transcriptome data of *C. wenyujin,* utilizing TB tools v1.09’ simple HMMER search with E-value ≤ 1e^−5^ (Chen et al., 2020; Khaksar and Sirikantaramas, 2021). The transcriptome data of *C. wenyujin* (Accession No. CRA000632) was available in the Genome Sequence Archive (GSA) database (https://ngdc.cncb.ac.cn/gsa/). The redundant sequences were removed utilizing the R program with a cutoff value of identity percentage > = 80%. MYB DBD of identified protein sequences was confirmed using online software SMART version 9 (http://smart.embl-heidelberg.de) and sequence search tool Pfam 35.0 (http://pfam.xfam.org/search/sequence) with default parameters. The identified MYB proteins were divided into four subfamilies based on the number of “R” in MYB DBD. ClustalX 2.1 software was used to perform multiple sequence alignment of conserved MYB DBD (Larkin et al., 2007). The sequence logos of conserved MYB DBD were created with the online software Weblogo 2.8.2 (http://weblogo.berkeley.edu/logo.cgi). Molecular weight (Mw) and theoretical isoelectric point (pI) of MYBs were predicted using the online software “Compute pI/Mw tool” (https://web.expasy.org/compute_pi/). The subcellular location information was predicted utilizing the online software WOLF PSORT (https://wolfpsort.hgc.jp/).

### Phylogenetic Analysis and Conserved Motif Analysis

Multiple amino acids sequence alignments of CwMYBs were performed using the software ClustalW 2.1 with the default parameters. The phylogenetic tree was generated by the neighbor-joining (NJ) method with 1,000 bootstrap replicates in MEGA 7.0 (Kumar et al., 2016). The online tool MEME 5.4.1 program (http://meme-suite.org/index.html) was used to detect conserved motifs of CwMYBs with the following parameters: zero or one per sequence, motif width ranges of 6–60 amino acids, and 20 as the maximum number of motifs. The default values were used for the remaining options. Only motifs with an E-value of < 1e^−2^ were kept for further analysis. The map was accomplished with TB tools v1.09 (Chen et al., 2020).

### Expression Profiles Analysis of *MYB* Genes

The RNA-seq data (https://www.ncbi.nlm.nih.gov/sra/, CRA003702) of fresh leaf and tuber samples from mature plants was used to analyze the spatial and temporal expression profiles of *MYBs* in *C. wenyujin* (Chen et al., 2021). The RNA-seq data (https://www.ncbi.nlm.nih.gov/sra/, CRA006461) was used to analyze the expression profiles of *MYBs* in *C. wenyujin* leaves with MeJA treatment. Transcripts per million reads (TPM) were obtained from RNA-seq data. Heatmaps were generated from log2 based TPM values using TB tools v1.09’ heatmap illustrator (Chen et al., 2020). The differentially expressed genes (DEGs) were screened with the following parameters: TPM >0, |log2Fold chang| >1, and *p*-value < 0.05.

### RNA Extraction and qRT-PCR Analysis

Total RNA was extracted from different samples with an RNAprep Pure Extraction Kit (DP441, TIANGEN, Beijing, China). First-strand cDNA was synthesized with PrimeScript™RT reagent Kit with gDNA Eraser (Takara, China). The CFX96 Touch™ Real-Time PCR Detection System (Bio-Rad, CA, United States) was used to perform quantitative real-time polymerase chain reaction (qRT-PCR) using ChamQ Universal SYBR qPCR Master Mix (Vazyme, Nanjing, China). The program parameters were as follows: 30 s at 95°C, 45-cycles of 10 s at 95°C, 30 s at 58°C, and 30 s at 72°C, and then 65–95°C for melting curve detection. Expression data was analyzed with the comparative 2^−ΔΔCt^ method (Livak and Schmittgen, 2001). Statistical analysis was performed using SPSS v17.0 software (IBM, New York, US). Statistical significance was set at ^*^ *p* < 0.05 and ^**^ *p* < 0.01. 18S rRNA was used as the internal control. The primers in this assay were listed in Supplementary Table S1.

### Yeast One-Hybrid Experiment

The Clontech Matchmaker™ Yeast One-Hybrid system (TBUSA, CA, United States), a GAL4-based yeast one-hybrid system, was employed to examine the transactivation activity. The complete open reading frames (ORF) and various truncated ORFs of *MYBs* were amplified by PCR (Supplementary Table S1). These fragments were then inserted into the pGBKT7 vector to construct corresponding recombinant plasmids. The plasmid pGBKT7 was used as a negative control. All of these plasmids were transformed into the yeast strain AH109, respectively. The transformation and screening were carried out following the users’ manual (Clontech, United States).

## Results

### Identification and Classification of *CwMYBs* in *C. wenyujin*

**FIGURE 1:**
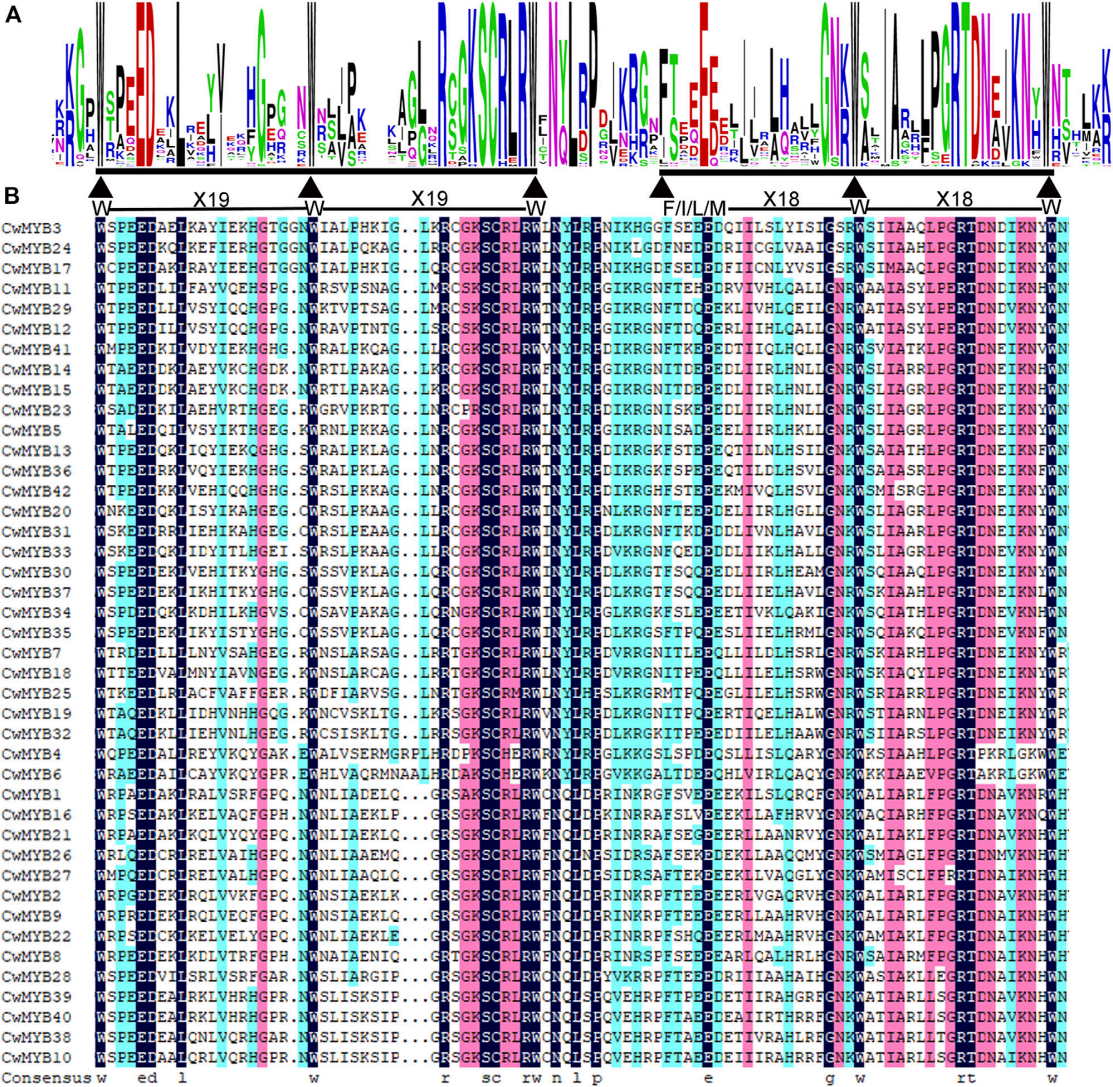
Conserved domains in R2R3 type CwMYB1-42 from *C. wenyujin*. **(A)** The sequence logos of conserved domains R2 and R3 repeat. Letter height indicates the sequence’s content and conservation at the corresponding position. Triangles in the MYB domain represent conserved amino acid residues or motifs. Weblogo 3 online software was used to create the image. **(B)** The conserved domain R2 (-W-X_19_-W-X_19_-W-) and R3 (-F/I/L/M-X_18_-W-X_18_-W-) repeats. The amino acid residues with a black background indicate 100% identity in the CwMYB1-42 protein sequences.

**FIGURE 2:**
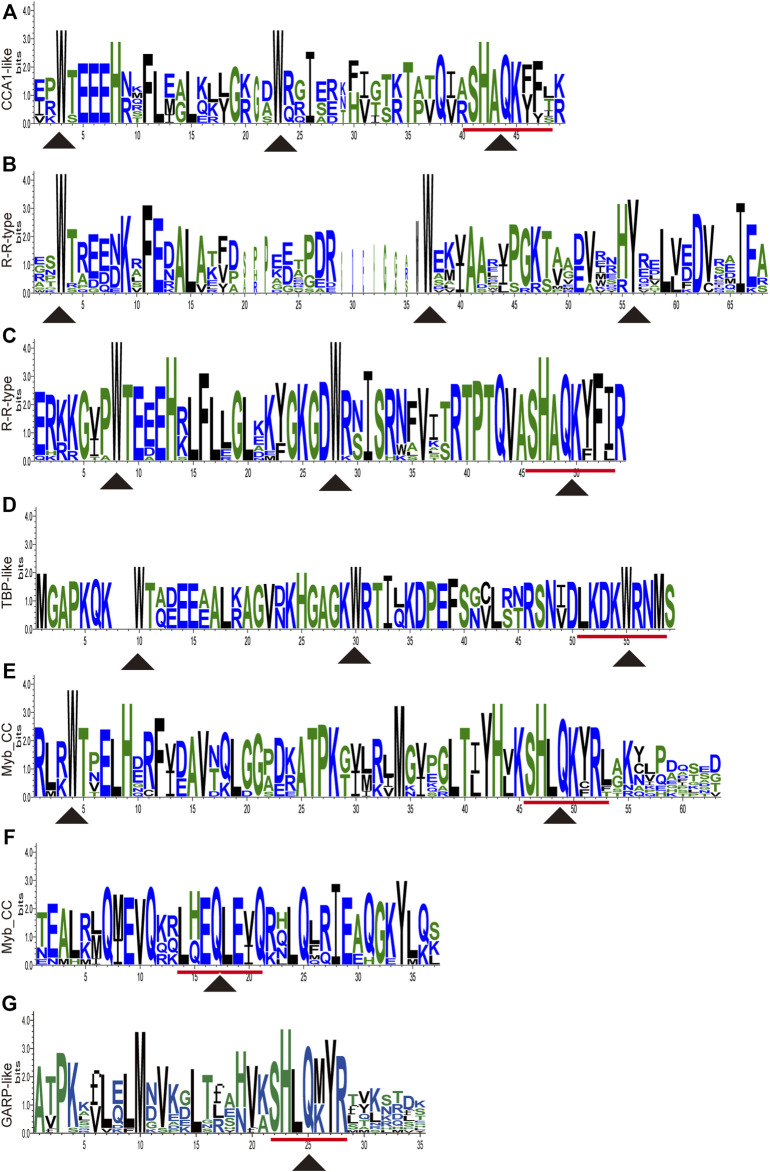
Sequence logos of conserved domains in MYB-related proteins from *C. wenyujin*. **(A)** CCA1-like type MYB-related proteins. **(B,C)** R-R type MYB-related proteins. **(D)** TBP-like type MYB-related proteins. **(E,F)** Myb_CC type MYB-related proteins. **(G)** GARP-like type MYB-related proteins. *Y* axis indicates the sequence content and its conservation at the corresponding position. Triangles indicate conserved amino acid residues or motifs in the MYB domain. The picture was drawn by TB tools software.

All the nucleotides and amino acid sequences of 88 *CwMYBs* identified from *C. wenyujin*, were listed in Supplementary Tables S2–S4. The MYB proteins were classified into four subfamilies based on the number of “R” in conserved MYB-DBD (Table 1). Forty-two CwMYBs containing typically R2 (-W-x19-W-x19-W-) and R3 (-F/I/L/M-x18-W-x18-W-) in conserved MYB-DBD were classified into the typical R2R3-MYB subfamily and named CwMYB1-42 (Figure 1). Two CwMYBs containing three “R” and one CwMYB containing four “R” in conserved MYB-DBD were classified into 3R-MYB subfamily and 4R-MYB subfamily respectively, referred as CwMYB3R1-2 and CwMYB4R1. A total of forty-three CwMYBs were classified into MYB-related subfamily with a single or partial “R” in MYB DBD, and named CwMYBR1-43. MYB-related subfamily was further divided into different groups due to the variable of “R” in MYB DBD. CwMYBR1-6, which contained the motif SHAQK (y/f)F in the “R” of the conserved MYB DBD, belonged to the CCA1-like group (Figure 2A). Two “R” were located in the N-terminus and middle of the sequences in CwMYBR7-19 respectively, separated by a long distance. The third tryptophan residue (W) in the first “R” was replaced by tyrosine residue (Y) (Figure 2B). The second “R” contained the motif SHAQK (y/f)F (Figure 2C). They are classified into R-R-type group in MYB-related subfamily. CwMYBR20 and CwMYBR21 were members of TBP-like group with a motif LKDKW(R/K) (N/T) in “R” of MYB DBD (Figure 2D). Based on a motif SHLQK/MxR in MYB DBD and the other domain Myb_CC_LHEQLE (pfam14379) in the no-MYB region, CwMYBR22-30 were identified as Myb-CC type MYB-related proteins (Figures 2E,F). Because of the variant MYB domain with motif SHLQK/MxR, CwMYBR31-43 were classified into GAPR-like group (Figure 2G). The predicted MYB proteins’ amino acid sequences ranged from 172 (CwMYB1) to 1,054 (CwMYB3R1), with the molecular weights ranged from 19.39 kDa (CwMYB1) to 115.44 kDa (CwMYB3R1). The theoretical isoelectric point (pI) ranged from 5.01 (CwMYB3R1) to 9.92 (CwMYBR12). The computed parameters of identified CwMYBs including Mw, pI, and subcellular localization were listed in Supplementary Table S5.

**TABLE 1:** Summary of the total identified *CwMYBs* in *C. wenyujin*.

*MYB* subfamily	Subgroup	Number of *MYBs*
*MYB*-related subfamily	CCA1-like	6
	R-R type	13
	TBP-like	2
	Myb_CC	9
	GARP-like	13
*R2R3-MYB* subfamily		42
*3R-MYB* subfamily		2
*4R-MYB* subfamily		1

### Phylogenetic and Motif Composition Analysis of MYB Proteins in *C. wenyujin*

**FIGURE 3:**
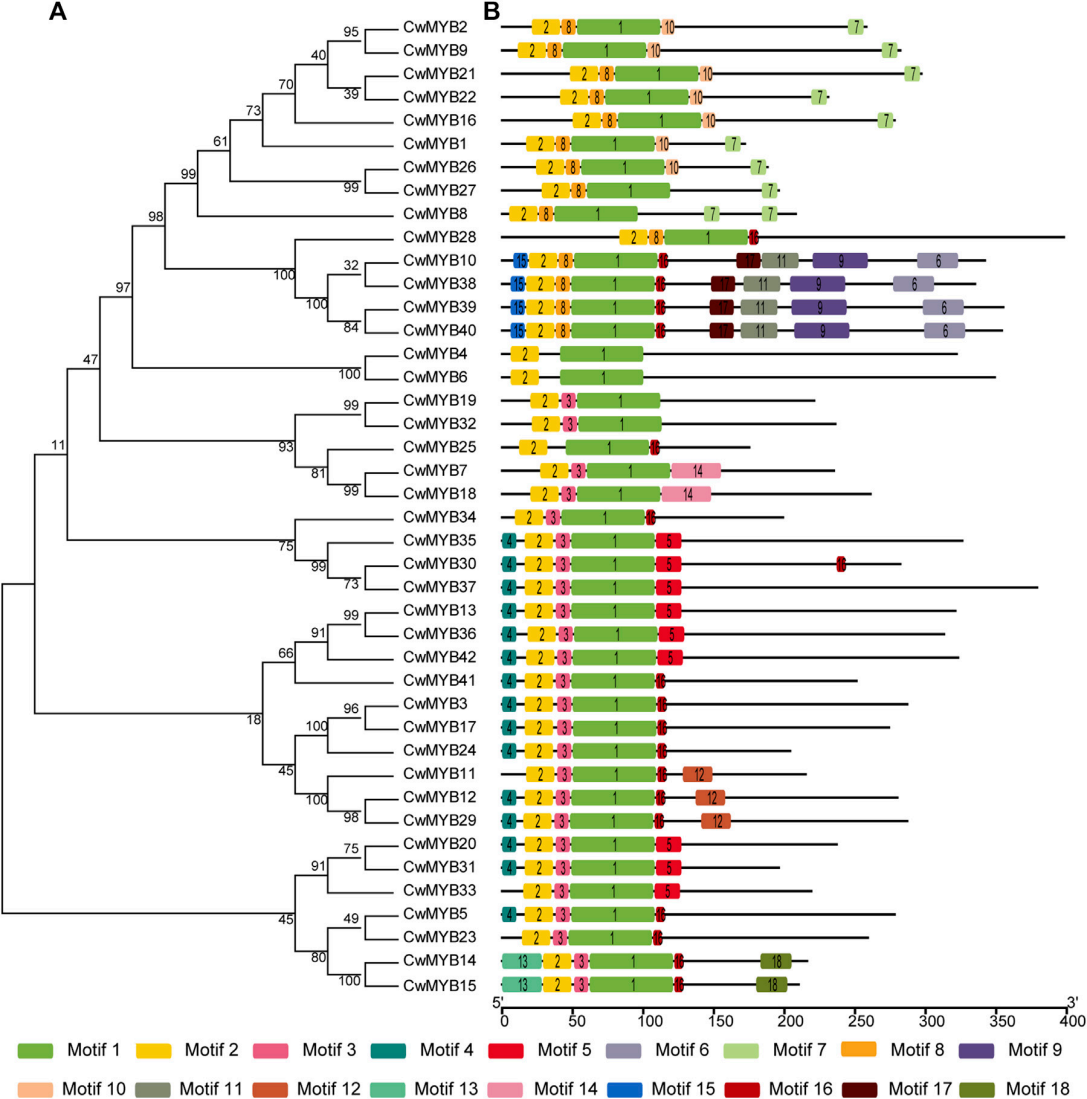
Phylogenetic tree and motif compositions of R2R3 type CwMYB1-42 proteins in *C. wenyujin*. **(A)** A phylogenetic tree of CwMYB1-42 was constructed using MEGA 7 software coupled with the Neighbor-Joining method with a bootstrap of 1,000 replicates. **(B)** Schematic diagrams of motif compositions. Colored boxes indicate different motifs. The picture was drawn by TB tools software.

In order to investigate the evolutionary relationship of CwMYB proteins in *C. wenyujin*, phylogenetic tree was constructed with the software MEGA 7.0 (Supplementary Figure S1). R2R3-MYBs, R-related MYBs, and 4R-MYBs were divided into different groups, whereas 3R-MYBs were divided into R2R3-MYBs group, indicating a closer evolutionary relationship. This result supports the hypothesis: 3R-MYBs evolve from R2R3-MYBs by acquiring an “R1”, or R2R3-MYBs evolve from 3R-MYBs by losing an “R1” (Jiang et al., 2004). Meanwhile, the phylogenetic relationship along with motif composition of R2R3-MYBs and R-related MYBs were also analyzed. The results revealed that the motifs distribution of MYB proteins supported the evolutionary relationship. Motif logos were illustrated in Supplementary Figures S2, S3. For R2R3-MYB proteins analysis (Figure 3), all MYB DBD contained motif 1 and motif 2, as well as motif 8 or motif 3 excepting CwMYB4/6/25. Non-MYB regions, outside of the MYB DBD, were highly variable and disordered, but similar in the same evolutionary cluster. Most CwMYBs contained one motif near MYB DBD such as motif 10/16/5. Besides, some CwMYBs had motifs located in the extreme C-terminal region such as CwMYB2/9/21/22/16/126/27/14/15, or throughout their non-MYB regions such as CwMYB10/38/39/40. It is worth noting that CwMYB10/38/39/40 contained a shorter EAR (LxLxL) motif in motif 9, which implies that this MYB may work as a repressor in gene transcription (Kagale and Rozwadowski, 2011).

**FIGURE 4:**
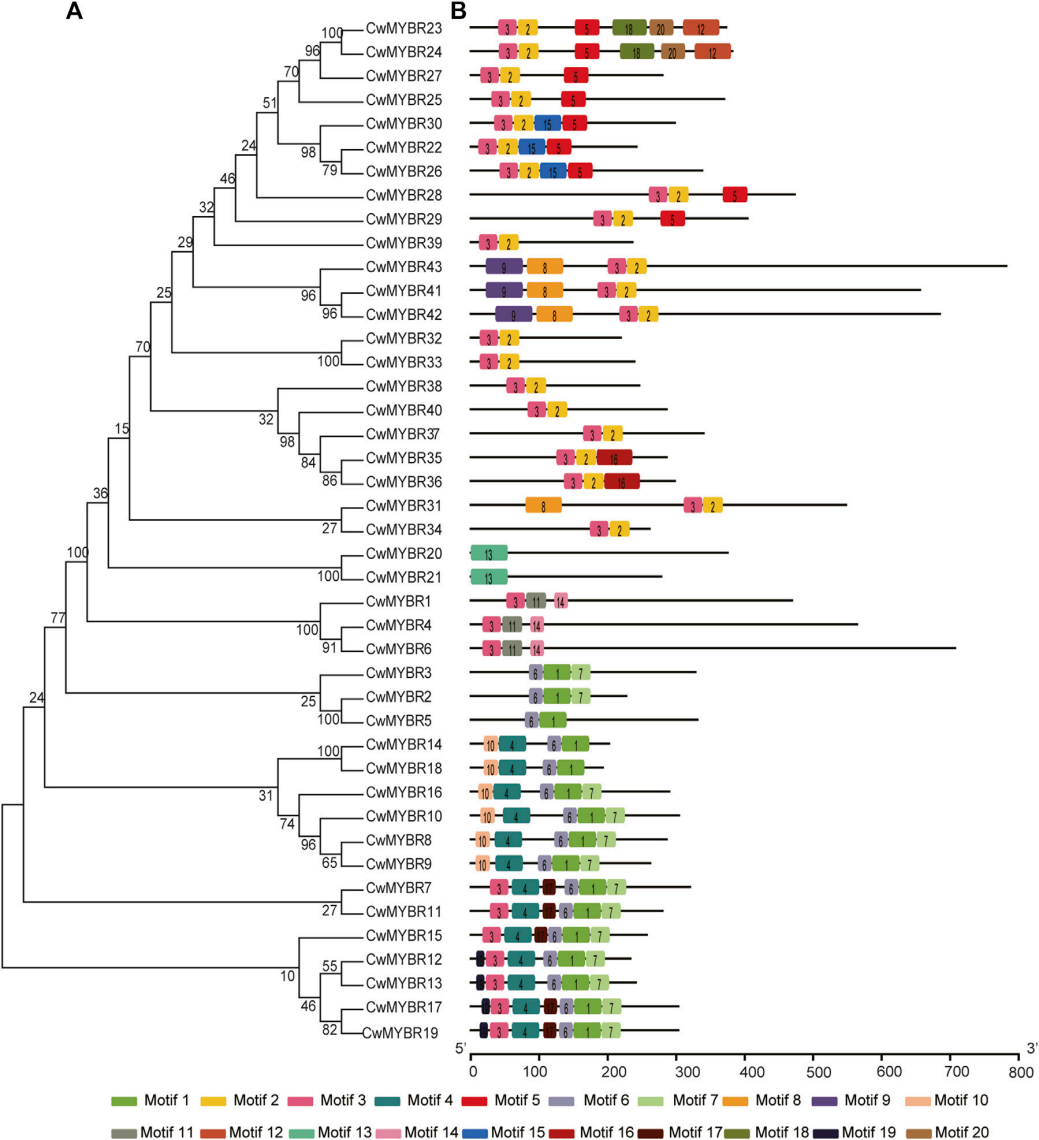
Phylogenetic tree and motif compositions of MYB-related proteins in *C. wenyujin*. **(A)** A phylogenetic tree of 43 MYB-related proteins was constructed by using MEGA 7 software coupled with Neighbor-Joining method with a bootstrap of 1,000 replicates. **(B)** Schematic diagrams of motif compositions. Colored boxes indicate different motifs. The picture was drawn by TB tools software.

MYB DBD domains of MYB-related proteins were less conservative than R2R3-MYBs, with an irregular distribution not always located in the N-terminal region (Figure 4). For example, CCA1-like MYB DBDs contained motifs 1/11 (CwMYBR1/4/6) or motifs 6/1 (CwMYBR2/3/5). The R-R-type MYB DBDs had motifs 10/4 (CwMYBR14/18/16/10/8/9) or motifs 3/4 (CwMYBR7/11/15/12/1317/19) in the first “R” structure, and motifs 6/1 in the second “R” structure, respectively. The MYB DBDs of Myb-CC type and GAPR-like MYBs were composed of motifs 3/2, while TBP-like MYB DBDs contained motif 13. Non-MYB regions outside of the MYB DBDs were also highly variable and disordered, same as R2R3-MYB proteins.

### Phylogeny of R2R3-MYBs Subfamily in Different Plants

**FIGURE 5:**
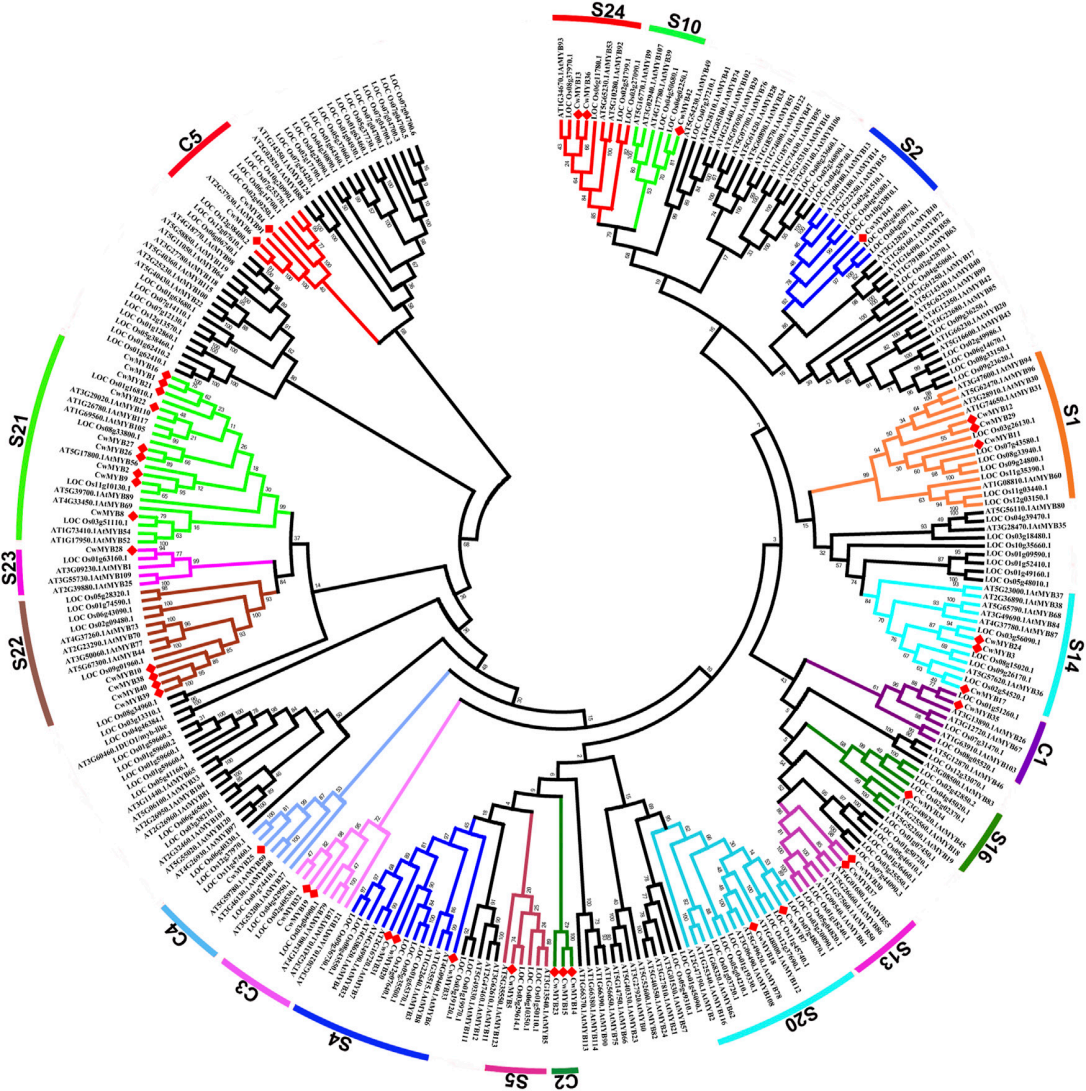
Phylogenetic tree of R2R3-MYBs in *C. wenyujin*, *Arabidopsis thaliana*, and *Oryza sativa*. The sequences contain 42 CwR2R3-MYBs in *C. wenyujin*, 126 AtR2R3-MYBs in Arabidopsis, and 130 OsR2R3-MYBs in rice. Forty-two full length CwMYB1-42 were divided into 13 subgroups (S) and 5 classes (C1-C5). The picture was generated using MEGA 7 software coupled with Neighbor-Joining method with a bootstrap of 1,000 replicates.

To understand the evolving relationship, the phylogenetic tree was constructed using R2R3-MYB proteins from *C. wenyujin*, *A. thaliana*, and *O. sativa* (Figure 5; Supplementary Table S6). Based on the conserved MYB DBD at the N-terminus and no-MYB regions at the C-terminus, AtMYBs had been classified into 23 subgroups (Dubos et al., 2010). According to the AtMYBs classification in Arabidopsis, thirty-six R2R3-MYBs of *C. wenyujin* were divided into 13 groups: S1, S2, S4, S5, S10, S13, S14, S16, S20, S21, S22, S23, and S24. Homologous genes in different plants are generally assumed to perform similar biological functions (Cai et al., 2012). CwMYB7/18 in subgroup 20, CwMYB10/38/39/40 in subgroup 22, and CwMYB41 in subgroup 2 could all play a role in abiotic stress responses. The evolutionary relationship of CwMYBs with AtMYBs and OsMYBs provided more valuable references for the functional prediction of CwMYBs in *C. wenyujin*. In addition, six R2R3-CwMYBs with AtMYBs and OsMYBs were distributed into four groups named C1, C3, C4, and C5, in which no MYBs have been characterized. It is worth noting that CwMYB14, CwMYB15, and CwMYB23 were classified into C2 which did not contain MYBs from Arabidopsis and rice, indicating the divergence of evolution in *C. wenyujin*, Arabidopsis, and rice.

### Expression Profiles of *CwMYBs* in Different Tissues

**FIGURE 6:**
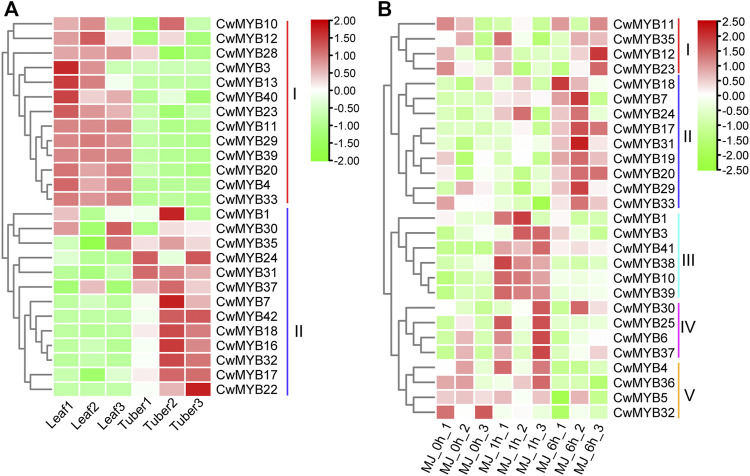
Expression profiles analysis of *R2R3*-*CwMYB* genes **(A)** *R2R3-CwMYB* gene expression patterns in tuber and leaf of *C. wenyujin*. **(B)** *R2R3*-*CwMYB* gene expression patterns in *C. wenyujin* with MeJA induction. Heatmap was created by TB tool using the transcript per million (TPM) values. The red and green cells represent the highest and lowest expression levels, respectively.

To investigate the expression patterns of *MYB* family genes in tuber and leaf, a heatmap was constructed based on TPM values from reported RNA-seq data. Finally, the expression patterns of 26 *R2R3-MYB* genes and 38 *MYB*-related genes were generated (Figure 6A; Supplementary Figure S4; Supplementary Table S7). The results revealed that the expression level of *R2R3-MYBs* in group I was higher in the leaf than that in the tuber. In contrast, *R2R3-MYBs* expression was lower in the leaf than that in the tuber in group II. *MYB*-related genes in group IV were expressed more in the tuber than in the leaf, whereas *MYB*-related genes in group VI were expressed less in the tuber than in the leaf. Differential expression analysis with strict standards revealed that 9 *R2R3-MYB* genes and 19 *MYB*-related genes were expressed in the tuber and leaf with significant difference (Supplementary Table S7).

### Expression Profiles of *CwMYB* Genes With MeJA Treatment

To explore the expression patterns of *MYB* family genes with MeJA treatment, a heatmap was constructed based on TPM values from reported RNA-seq data. Finally, the expression patterns of 27 *R2R3-CwMYB* genes and 28 *MYB*-related genes were generated. The expression analysis results of *R2R3-CwMYB* genes revealed that the expression profiles in groups II, III, and IV were up-regulated (Figure 6B). *R2R3-MYB* expression levels were upregulated 1 h after MeJA induction in groups III and IV, while 6 h after MeJA induction in group II. Instead, with 1 and 6 h of MeJA treatment, the expression levels of *R2R3-MYBs* were down-regulated in groups I and V respectively. In the expression analysis of *MYB*-related genes (Supplementary Figure S5), the expression profiles in groups I and II were upregulated after 6 and 1 h of MeJA induction, respectively. Concurrently, the expression profiles of *MYB* genes were downregulated in groups III and IV. Differential expression analysis with strict standards revealed that 5 *R2R3-MYB* genes and 8 *MYB*-related genes were involved in the response to MeJA treatment (Supplementary Table S8).

### The Expression Levels and Transcriptional Activation Analysis of Four Candidate *MYBs* in *C. wenyujin*

**FIGURE 7:**
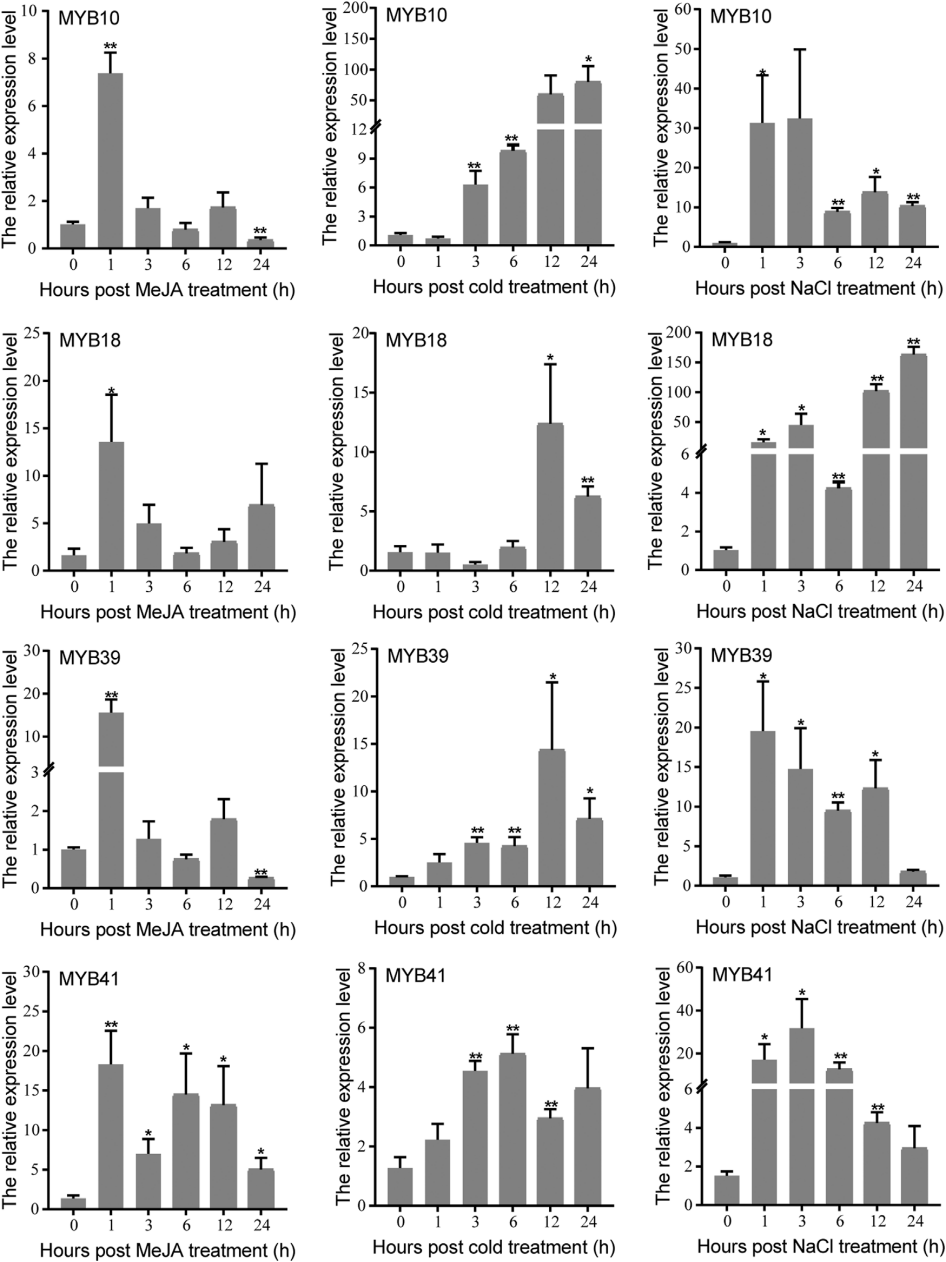
Expression levels of four candidate R2R3-type *CwMYB*s under cold, NaCl, and MeJA treatments. Three biological replicates were performed. Vertical bars refer to ±SE (*n* = 3). Asterisks indicate significant differences (^*^ *p* < 0.05; ^**^ *p* < 0.01).

The MYB protein is well known for its role in plant response to various abiotic stresses. In this study, candidate *MYB* genes *CwMYB10/18/39/41* were screened based on the results of phylogeny and expression analysis. The expression levels of four candidate *CwMYBs* were determined using qRT-PCR with cold, NaCl and MeJA treatments. According to the findings, expression levels of all four *MYB* genes were significantly increased by cold, NaCl, and MeJA treatments (Figure 7). As a result, these MYB proteins may act as positive regulators in *C. wenyujin* in response to various abiotic stresses.

**FIGURE 8:**
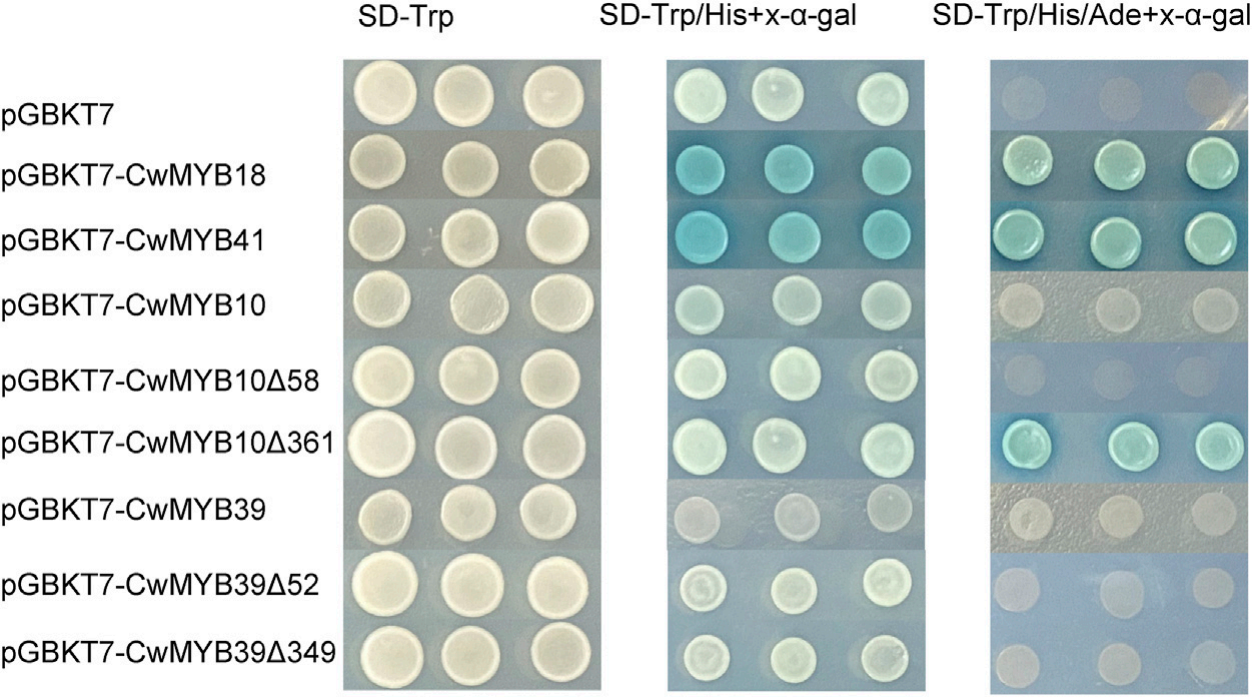
Analysis of the transactivation activity of four candidates R2R3-type CwMYBs in yeast. Recombinant plasmids were transformed into yeast strain AH109, and the transformant strains were screened by SD/−Trp, SD/−Trp/−His + X-a-gal, and SD/−Trp/−His/−Ade + Xa-gal media.

To investigate the transactivation activity of MYB TFs, the yeast one-hybrid experiment was performed. The complete and various truncated ORFs of *MYB* were cloned into the pGBKT7 plasmid to obtain recombinant plasmids GAL4BD-*CwMYB*. Then recombinant plasmids were transformed into the yeast strain AH109 respectively, to examine the transactivation ability (Figure 8). The recombinant strains GAL4BD-*CwMYB18* and GAL4BD-*CwMYB41* grew well and turned blue on SD-Trp/His/Ade medium with X-α-galactoside (X-α-gal), while recombinant strains GAL4BD-*CwMYB10* and GAL4BD-*CwMYB39* cannot develop normally. The recombinant strains harbouring truncated *CwMYB10Δ361* (only with the C-terminus of *CwMYB10*) grew well and turned blue on SD-Trp/His/Ade medium with X-α-gal. The recombinant strains harbouring various truncated *CwMYB39* remained unable to grow on SD-Trp/His/Ade medium with X-α-gal. The results confirmed the activity of transcriptional activation for complete *CwMYB18*, *CwMYB41* and truncated *CwMYB10Δ361*, but not for complete *CwMYB10*, *CwMYB39* and truncated *CwMYB39*.

## Discussion

Plants are frequently subjected to harsher environments. After long-term evolution, they have evolved highly efficient mechanisms to adapt to stress conditions. Plants TFs function as key regulators of gene expression networks, controlling various developmental and physiological processes in plants (Meraj et al., 2020; Manna et al., 2021; Romani and Moreno, 2021). MYB family is one of the largest transcription factor families in plants. The large size of MYB family reflects their importance and functional diversity in regulating plant physiological and biochemical processes. Previous studies depicted that MYBs are involved in plant development, cell shape and petal morphogenesis, cellular proliferation and differentiation, trichome development, phenylpropanoid metabolism, hormone responses, biotic and abiotic stress responses, etc (Baillo et al., 2019; Cao et al., 2020; Wang et al., 2021; Xiao et al., 2021).

Nevertheless, *MYBs* in *C. wenyujin* are entirely unknown to date. Studies on the identification and characterization of *MYBs* would provide insights into the understanding of *CwMYB* family and their response to abiotic stresses in *C. wenyujin*. Based on RNA-seq data, 88 *CwMYBs* were identified from *C. wenyujin* for the first time, including 43 *MYB*-related genes, 42 *R2R3-MYB* genes, two *3R-MYB* genes, and one *4R-MYB* genes (Table 1 and Supplementary Tables S2–S5). The identification of *CwMYB* superfamily members may be limited due to the lackness of *C. wenyujin* genome data. The genome sequencing of *C. wenyujin* in the future will be a significant step forward in comprehensively understanding of the *CwMYB* superfamily.

The domains and motifs of transcription factors are often associated with transcriptional activity, protein-protein interactions, and DNA binding (Millard et al., 2019). R2R3-MYB proteins featured a highly conserved MYB DBD at the N-terminus containing “R2” and “R3” structures. The first tryptophan (W) of “R3” was frequently replaced by phenylalanine (F), isoleucine (I), leucine (L), or methionine (M). However, the three tryptophans (W) of “R2” were highly conserved (Ambawat et al., 2013) (Figure 1). The MYB DBD of MYB-related protein was variable and randomly distributed not always located in the N-terminal region. The third tryptophan (W) of “R” structure is often replaced by specific motif, such as SHAQK (y/f)F, LKDKW(R/K) (N/T), SHLQK/MxR, or Myb_CC_LHEQLE, which is the criterion for MYB subfamily grouping (Du et al., 2013) (Figure 2). Different MYB DBDs have variations in the DNA-binding preferences, but it’s highly similar for DNA-binding specificity of MYBs with close evolutionary relationships. It suggests that other properties of proteins contribute to functional differentiation (Millard et al., 2019). Non-MYB regions are essential for explaining the highly functional diversity because of the more significant sequence diversity than MYB DBDs. Motif composition analysis of CwMYB proteins revealed differences of motifs in the non-MYB regions, indicating the functional diversity (Figures 3, 4). As we all know, protein function is closely related to its structure. Unfortunately, most of these conserved motifs are orphan and have not been linked to specific molecular functions yet (Millard et al., 2019).

The *R2R3-MYB* subfamily is the largest one in *MYB* superfamily, and the corresponding function research is also comprehensive and clear in various plants (Ambawat et al., 2013). Many studies suggest that the homologous MYB TFs in different species may play similar roles. The functional research of MYB proteins on model plants such as Arabidopsis and rice, is relatively clear. In Arabidopsis, 126 *R2R3-MYBs* have been classified into 23 subgroups based on the no-MYB regions of C-terminal. The members in the same subgroup play a similar regulatory role in plant growth and development processes. In subgroup 2, AtMYB14 and AtMYB15 were reported to regulate cold tolerance in Arabidopsis by affecting expression of *CBF* genes (Agarwal et al., 2006; Chen et al., 2013). In subgroup 20, AtMYB62, AtMYB112, AtMYB2, and AtMYB108 are involved in response to drought, salinity, high light, and Pi-starvation stresses through hormone dependent or independent mechanisms in Arabidopsis (Devaiah et al., 2009; Cui et al., 2013; Lotkowska et al., 2015; Jia et al., 2020). In subgroup 22, AtMYB44 participates in various hormone mediated responses to biotic and abiotic stress in Arabidopsis (Nguyen and Cheong, 2018; Nguyen et al., 2019). AtMYB73 is a negative regulator of salt overly sensitive (*SOS*) gene. Loss of *AtMYB73* causes hyper-induction of the *SOS1* and *SOS3* genes in response to high salinity in Arabidopsis (Kim et al., 2013). According to phylogenetic relationships of *CwMYBs* and *AtMYBs* (Figure 5), it was speculated that the homologous genes *CwMYB41* in subgroup 2, *CwMYB10* and *CwMYB39* in subgroup 22, as well as *CwMYB18* in subgroup 20 may play roles in abiotic and biotic stress responses in *C. wenyujin*. Besides, the significantly increased expression level of *CwMYB10*, *CwMYB39,* and *CwMYB41* under MeJA stress further indicated that they may play roles as transcriptional regulators (Figure 6). A subsequent experiment revealed that *CwMYB10*, *CwMYB18*, *CwMYB39*, and *CwMYB41* were significantly induced with different expression patterns under cold, NaCl, and MeJA treatments (Figure 7). The activity of transcriptional activation was confirmed for complete *CwMYB18* and *CwMYB41*, while not for complete *CwMYB39* and *CwMYB10*. Therefore, CwMYB18 and CwMYB41 proteins participated in transcriptional regulation as transcriptional activators in *C. wenyujin*. Furthermore, truncated *CwMYB10Δ361* lacking the N-terminus exhibited transcriptional activation activity, whereas all kinds of truncated *CwMYB39* did not (Figure 8). Motif analysis revealed that CwMYB39 and CwMYB10 proteins contained an EAR motif (LxLxL), which implies that this MYB may work as a repressor in gene transcription (Kagale and Rozwadowski, 2011). CwMYB39 and CwMYB10 belonging to subgroup 22, have a close evolutionary relationship with AtMYB44 and AtMYB73 that have been thoroughly studied. Complete AtMYB44 and EAR (LxLxL) motif-mutated *Atmyb44* have no activity of transcriptional self-activating. AtMYB44 functions as a repressor of gene transcription in stress responses. For example, AtMYB44 interacts with TOPLESS-RELATED (TPR) corepressors to repress negative regulators PP2Cs, thus playing a positive regulator in the ABA-mediated stress responses (Nguyen and Cheong, 2018). AtMYB44 represses the transcription of drought response gene *AtLEA4-5* under normal conditions, but is eliminated in response to osmotic stress (Nguyen et al., 2019). Not coincidentally, AtMyb73 acts as a negative regulator in response to salt stress by repressing *SOS* induction in Arabidopsis (Kim et al., 2013). Therefore, the regulation of CwMYB10 and CwMYB39 in response to abiotic stresses as the transcription repressors may rely on their modification or interaction with other cofactors.

In conclusion, the first transcriptome-wide identification and analysis of *CwMYBs* in *C. wenyujin* will serve as a foundation for future functional characterization. Following the successful construction of the *C. wenyujin* genetic transformation system, the future research will focus on the functional characterization of candidate regulators CwMYB10, CwMYB18, CwMYB39, and CwMYB41.

## Data Availability

The datasets presented in this study can be found in online repositories. The names of the repository/repositories and accession number(s) can be found in the article/Supplementary Material.
