# Supplementary material for: Identification and Analysis of MYB Gene Family for Discovering Potential Regulators Responding to Abiotic Stresses in Curcuma wenyujin
Source: Front Genet. 2022 Apr 25;13:894928. doi: 10.3389/fgene.2022.894928 (PMC9081655; doi:10.3389/fgene.2022.894928)
Supplement: Supplementary file 1 [file DataSheet1.ZIP › Table S5.docx]

Table S5 Physical parameters of CwMYBs in *C. wenyujin*

| **CwMYBs** | **Subfamily** | **Mw (Da)** | **pI** | **Subcellular localization** |
| --- | --- | --- | --- | --- |
| CwMYBR1 | MYB-related | 52223.29 | 6.16 | Nucleus |
| CwMYBR2 | MYB-related | 24986.37 | 9.3 | Nucleus |
| CwMYBR3 | MYB-related | 35517.94 | 7.86 | Nucleus |
| CwMYBR4 | MYB-related | 60906.78 | 8.28 | Nucleus |
| CwMYBR5 | MYB-related | 35921.67 | 9.1 | Chloroplast |
| CwMYBR6 | MYB-related | 78140.46 | 6.16 | Nucleus |
| CwMYBR7 | MYB-related | 35926.51 | 9.05 | Chloroplast |
| CwMYBR8 | MYB-related | 30760.32 | 9.37 | Nucleus |
| CwMYBR9 | MYB-related | 28687.29 | 9.51 | Cytoplasm |
| CwMYBR10 | MYB-related | 32925.72 | 6.96 | Nucleus |
| CwMYBR11 | MYB-related | 30857.86 | 8.86 | Nucleus |
| CwMYBR12 | MYB-related | 26033.52 | 9.92 | Cytoplasm |
| CwMYBR13 | MYB-related | 26721.62 | 7.82 | Nucleus |
| CwMYBR14 | MYB-related | 22768.16 | 5.59 | Nucleus |
| CwMYBR15 | MYB-related | 29167.73 | 9.07 | Nucleus |
| CwMYBR16 | MYB-related | 30714.15 | 6.51 | Cytoskeleton |
| CwMYBR17 | MYB-related | 33618.47 | 6.33 | Nucleus |
| CwMYBR18 | MYB-related | 22115.55 | 8.66 | Nucleus |
| CwMYBR19 | MYB-related | 34502.23 | 5.37 | Nucleus |
| CwMYBR20 | MYB-related | 41885.27 | 9.41 | Nucleus |
| CwMYBR21 | MYB-related | 30692.94 | 9.48 | Nucleus |
| CwMYBR22 | MYB-related | 26587.42 | 5.34 | Nucleus |
| CwMYBR23 | MYB-related | 41610.68 | 7.68 | Nucleus |
| CwMYBR24 | MYB-related | 42561.79 | 7.01 | Nucleus |
| CwMYBR25 | MYB-related | 40703.46 | 8.93 | Nucleus |
| CwMYBR26 | MYB-related | 36474.49 | 6.37 | Nucleus |
| CwMYBR27 | MYB-related | 30422.37 | 5.8 | Nucleus |
| CwMYBR28 | MYB-related | 52446.6 | 5.83 | Nucleus |
| CwMYBR29 | MYB-related | 44492.44 | 5.35 | Nucleus |
| CwMYBR30 | MYB-related | 33247.19 | 6.32 | Nucleus |
| CwMYBR31 | MYB-related | 60845.75 | 5.89 | Peroxisome |
| CwMYBR32 | MYB-related | 25257.5 | 8.9 | Nucleus |
| CwMYBR33 | MYB-related | 27522.05 | 8.73 | Nucleus |
| CwMYBR34 | MYB-related | 29173.05 | 7.6 | Nucleus |
| CwMYBR35 | MYB-related | 31847.68 | 8.89 | Nucleus |
| CwMYBR36 | MYB-related | 33193.1 | 6.91 | Nucleus |
| CwMYBR37 | MYB-related | 37392.91 | 8.48 | Nucleus |
| CwMYBR38 | MYB-related | 27291.45 | 8.39 | Nucleus |
| CwMYBR39 | MYB-related | 26261.84 | 9.31 | Nucleus |
| CwMYBR40 | MYB-related | 32482.5 | 7.92 | Nucleus |
| CwMYBR41 | MYB-related | 71570.82 | 6.38 | Peroxisome |
| CwMYBR42 | MYB-related | 75350.73 | 5.66 | Nucleus |
| CwMYBR43 | MYB-related | 86810.1 | 6.24 | Nucleus |
| CwMYB1 | R2R3 | 19392.83 | 9.64 | Nucleus |
| CwMYB2 | R2R3 | 28823.35 | 9.45 | Nucleus |
| CwMYB3 | R2R3 | 32456.64 | 6.26 | Nucleus |
| CwMYB4 | R2R3 | 36623.33 | 9.45 | Nucleus |
| CwMYB5 | R2R3 | 30882.75 | 5.74 | Nucleus |
| CwMYB6 | R2R3 | 40005.62 | 9.39 | Nucleus |
| CwMYB7 | R2R3 | 27586.14 | 6.61 | Nucleus |
| CwMYB8 | R2R3 | 24133.91 | 7.81 | Nucleus |
| CwMYB9 | R2R3 | 32244.29 | 9.78 | Nucleus |
| CwMYB10 | R2R3 | 37264.58 | 6.31 | Nucleus |
| CwMYB11 | R2R3 | 24020.04 | 6.66 | Nucleus |
| CwMYB12 | R2R3 | 30927.57 | 5.9 | Nucleus |
| CwMYB13 | R2R3 | 35789.09 | 5.68 | Nucleus |
| CwMYB14 | R2R3 | 25242.68 | 8.28 | Nucleus |
| CwMYB15 | R2R3 | 24353.72 | 9.16 | Nucleus |
| CwMYB16 | R2R3 | 30587.06 | 7.15 | Nucleus |
| CwMYB17 | R2R3 | 31463.29 | 7.55 | Nucleus |
| CwMYB18 | R2R3 | 29537.16 | 5.17 | Nucleus |
| CwMYB19 | R2R3 | 26080.1 | 6.11 | Nucleus |
| CwMYB20 | R2R3 | 26699.06 | 8.4 | Nucleus |
| CwMYB21 | R2R3 | 33149.94 | 9.89 | Nucleus |
| CwMYB22 | R2R3 | 26223.6 | 7.68 | Nucleus |
| CwMYB23 | R2R3 | 29995.74 | 8.55 | Nucleus |
| CwMYB24 | R2R3 | 22723.08 | 9.16 | Nucleus |
| CwMYB25 | R2R3 | 20329.06 | 9.2 | Nucleus |
| CwMYB26 | R2R3 | 21407.11 | 9.15 | Nucleus |
| CwMYB27 | R2R3 | 22404.42 | 9.44 | Nucleus |
| CwMYB28 | R2R3 | 43209.5 | 7.59 | Nucleus |
| CwMYB29 | R2R3 | 31961.14 | 7.64 | Nucleus |
| CwMYB30 | R2R3 | 31238.27 | 7.17 | Nucleus |
| CwMYB31 | R2R3 | 22169.37 | 9.1 | Nucleus |
| CwMYB32 | R2R3 | 27085.19 | 5.9 | Nucleus |
| CwMYB33 | R2R3 | 24902.78 | 6.44 | Nucleus |
| CwMYB34 | R2R3 | 22794.68 | 8.94 | Nucleus |
| CwMYB35 | R2R3 | 35943.66 | 6.05 | Nucleus |
| CwMYB36 | R2R3 | 35349.73 | 6.79 | Nucleus |
| CwMYB37 | R2R3 | 40891.96 | 6.32 | Nucleus |
| CwMYB38 | R2R3 | 36269.88 | 9.02 | Nucleus |
| CwMYB39 | R2R3 | 38785.4 | 9.24 | Nucleus |
| CwMYB40 | R2R3 | 39004.81 | 8.82 | Nucleus |
| CwMYB41 | R2R3 | 28318.55 | 5.24 | Nucleus |
| CwMYB42 | R2R3 | 36260.04 | 6.41 | Nucleus |
| CwMYB3R1 | 3R | 115437.57 | 5.01 | Nucleus |
| CwMYB3R2 | 3R | 62464.83 | 8.46 | Nucleus |
| CwMYB4R1 | 4R | 109708.47 | 9.24 | Nucleus |
