# Supplementary material for: Identification and Analysis of MYB Gene Family for Discovering Potential Regulators Responding to Abiotic Stresses in Curcuma wenyujin
Source: Front Genet. 2022 Apr 25;13:894928. doi: 10.3389/fgene.2022.894928 (PMC9081655; doi:10.3389/fgene.2022.894928)

Motif 1

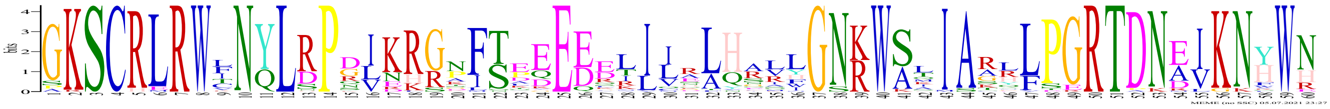

Motif 2

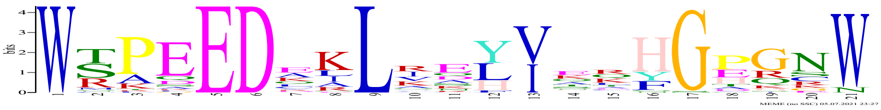

Motif 3

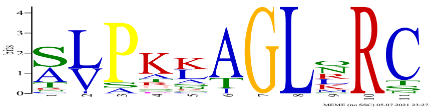

Motif 4

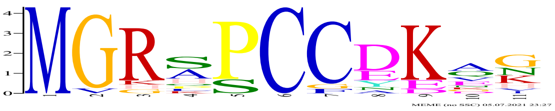

Motif 5

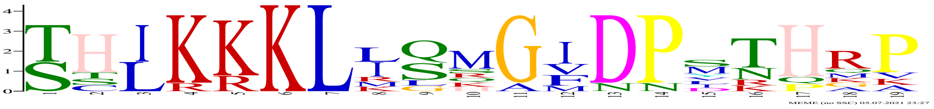

Motif 6

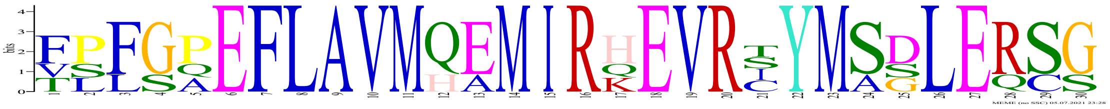

Motif 7

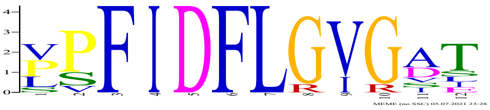

Motif 8

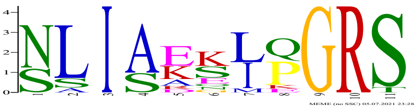

Motif 9

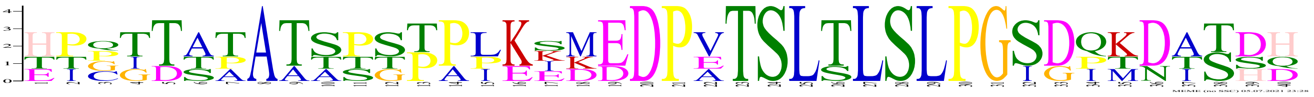

Motif 10

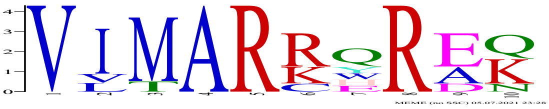

Motif 11

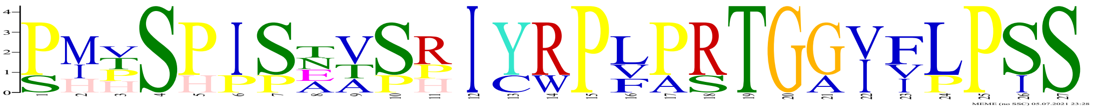

Motif 12

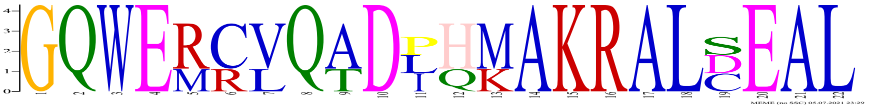

Motif 13

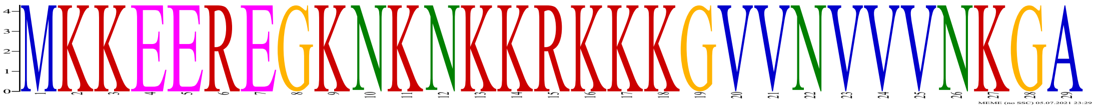

Motif 14

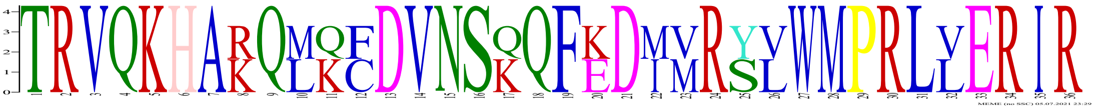

Motif 15

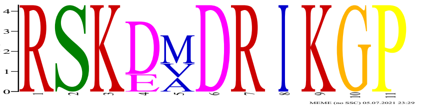

Motif 16

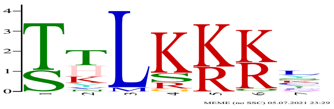

Motif 17

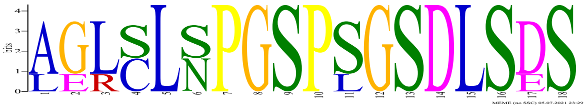

Motif 18

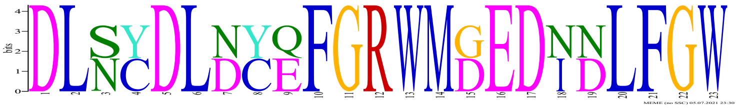

Supplement: Supplementary file 1 [file DataSheet1.ZIP › Figure S2 .pdf]
