# Supplementary figures and images for: Identification and Analysis of MYB Gene Family for Discovering Potential Regulators Responding to Abiotic Stresses in Curcuma wenyujin
Source: Front Genet. 2022 Apr 25;13:894928. doi: 10.3389/fgene.2022.894928 (PMC9081655; doi:10.3389/fgene.2022.894928)

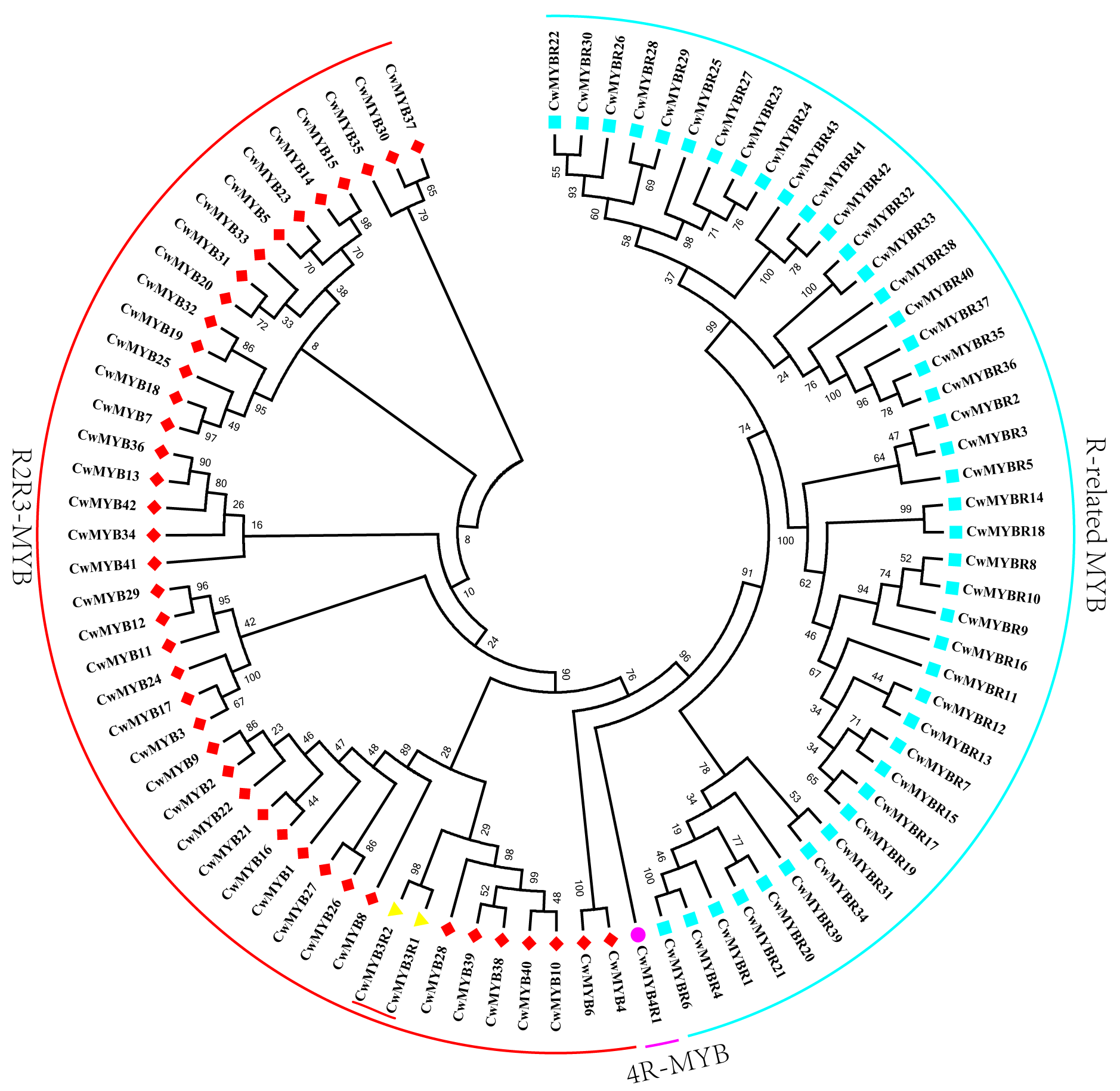

Supplement: Supplementary file 1 [file DataSheet1.ZIP › Figure S1.tif]

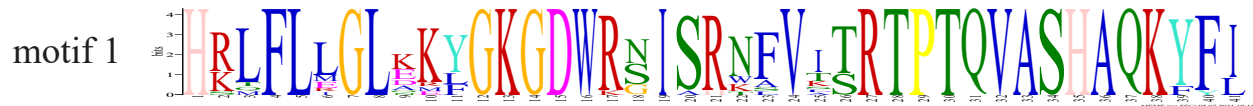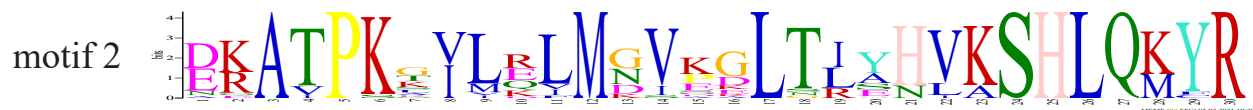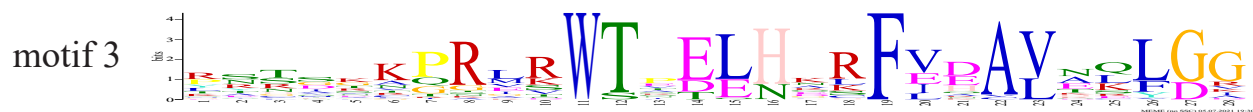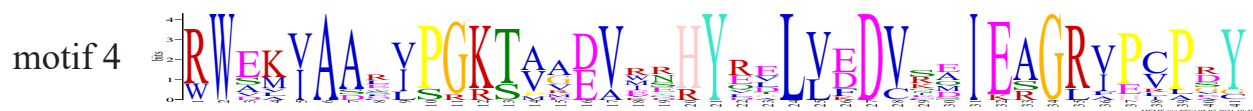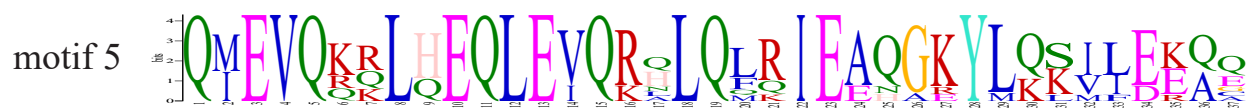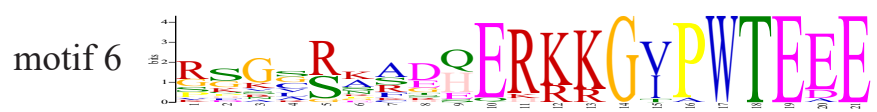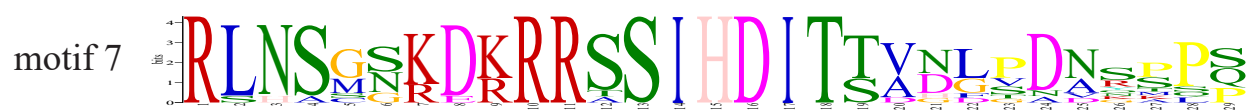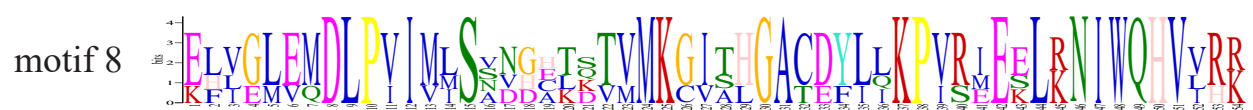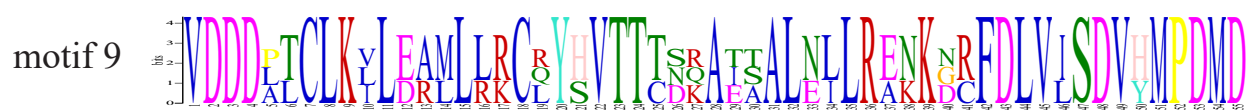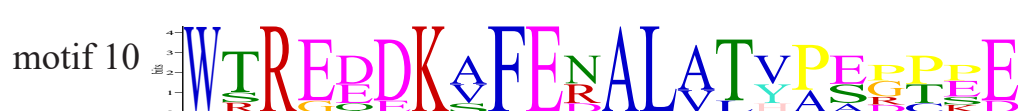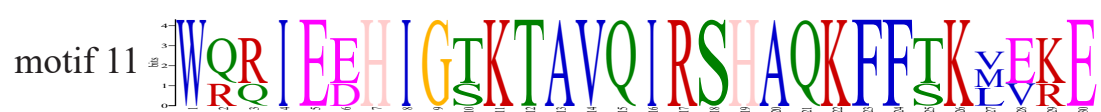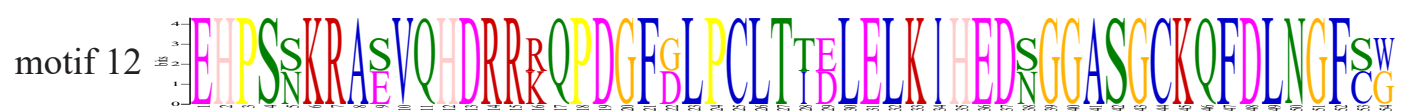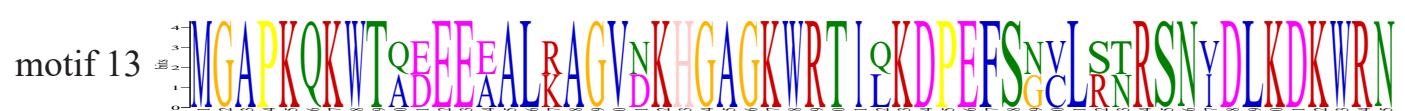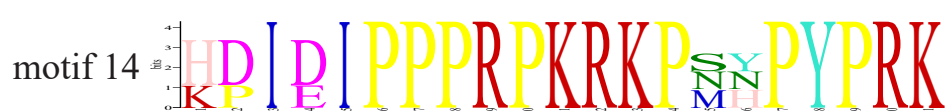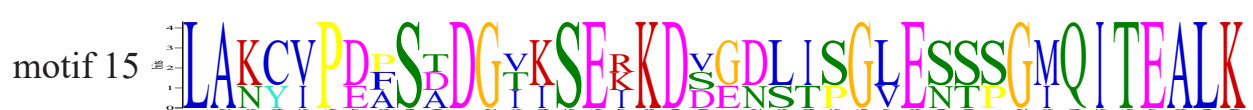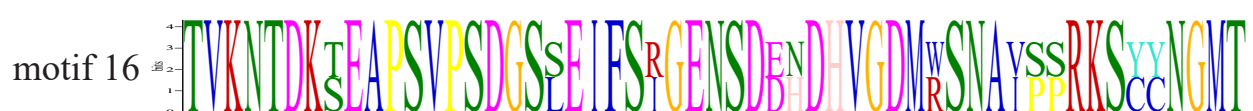

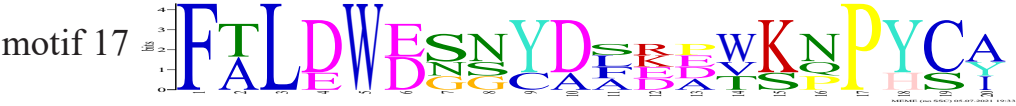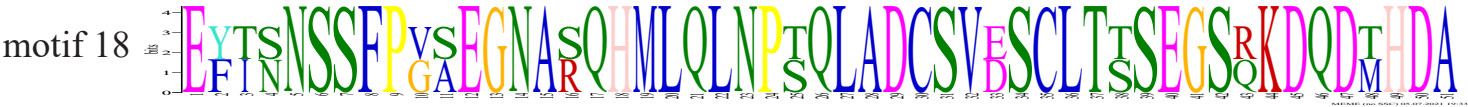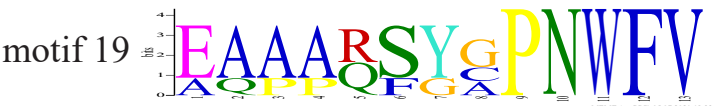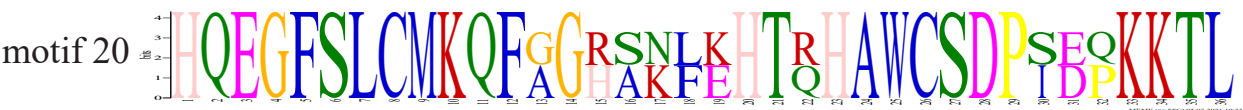

Supplement: Supplementary file 1 [file DataSheet1.ZIP › Figure S3.pdf]

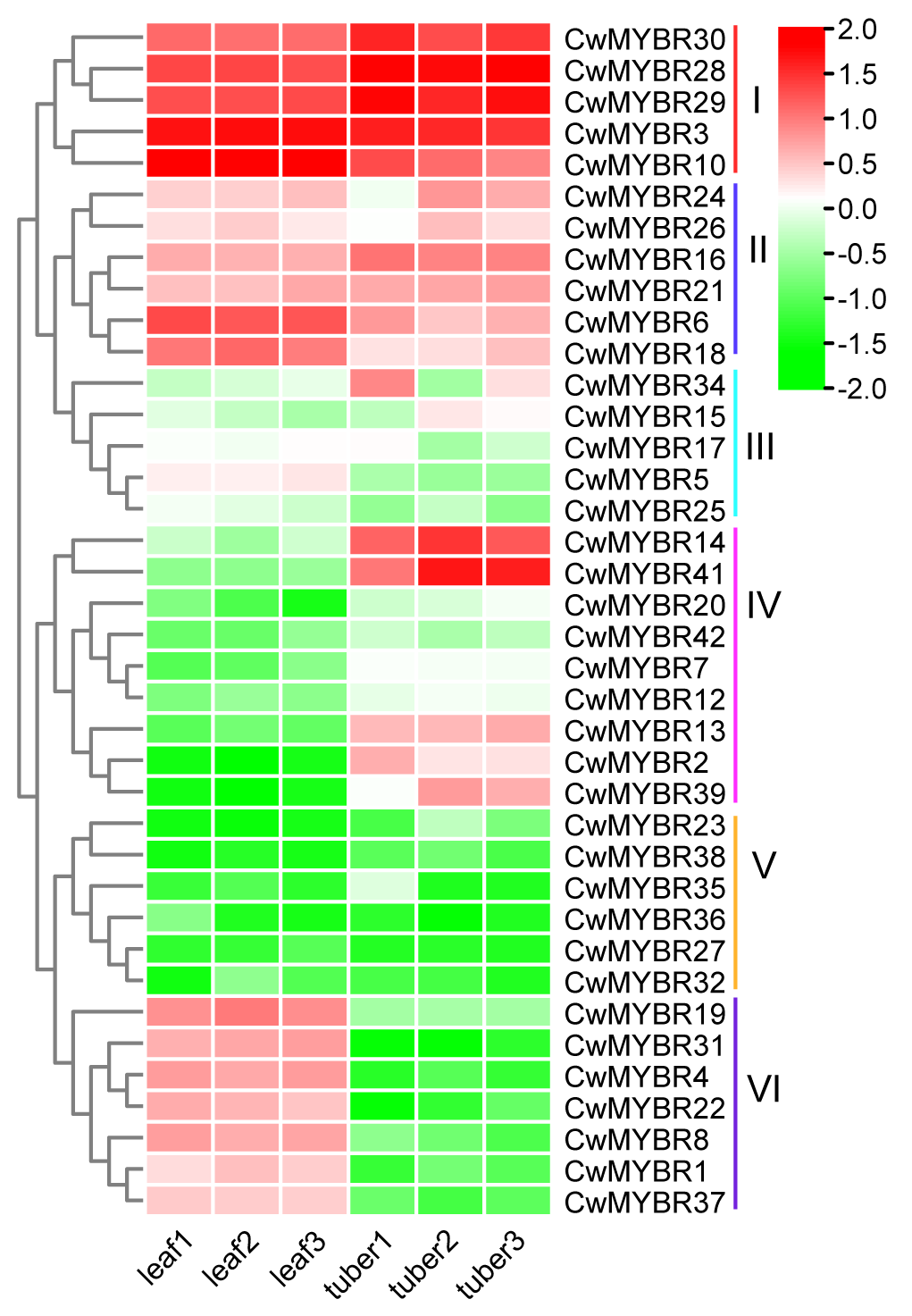

Supplement: Supplementary file 1 [file DataSheet1.ZIP › Figure S4.tif]

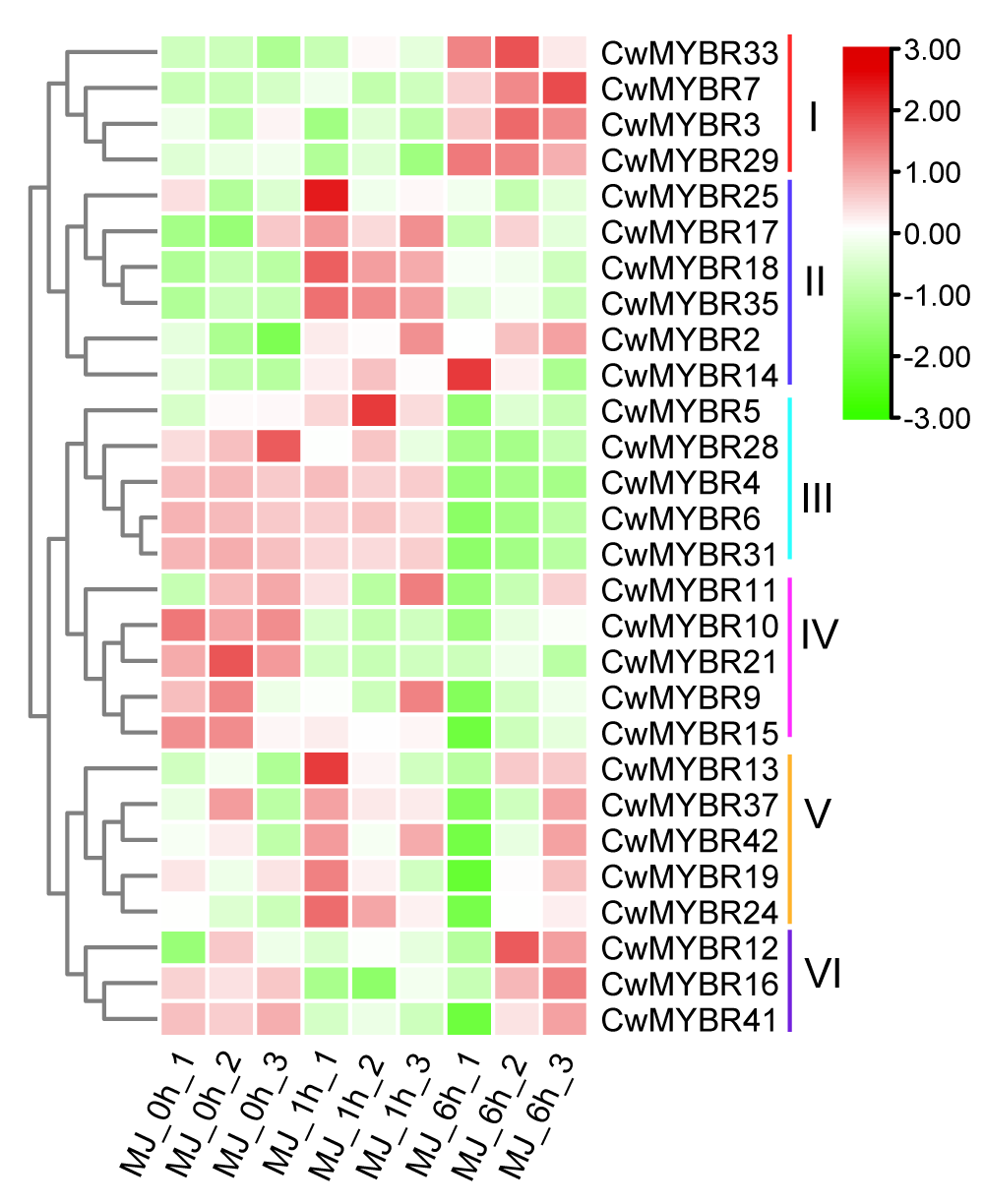

Supplement: Supplementary file 1 [file DataSheet1.ZIP › Figure S5.tif]
